# *Deltaproteobacteria* (*Pelobacter*) and *Methanococcoides* are responsible for choline-dependent methanogenesis in a coastal saltmarsh sediment

**DOI:** 10.1038/s41396-018-0269-8

**Published:** 2018-09-11

**Authors:** Eleanor Jameson, Jason Stephenson, Helen Jones, Andrew Millard, Anne-Kristin Kaster, Kevin J. Purdy, Ruth Airs, J. Colin Murrell, Yin Chen

**Affiliations:** 1 School of Life Sciences, University of Warwick, Warwick, CV4 7AL UK; 2 Department of Infection, Immunity and Inflammation, University of Leicester, Leicester, LE1 7RH UK; 3 Karlsruhe Institute of Technology, Institute for Biological Interfaces (IBG 5), Hermann-von-Helmholtz-Platz 1, Eggenstein-Leopoldshafen, Karlsruhe, 76344 Germany; 4 Plymouth Marine Laboratory, Prospect Place, The Hoe, Plymouth, PL1 3DH UK; 5 University of East Anglia, Norwich Research Park, Norfolk, Norwich, NR4 7TJ UK

**Keywords:** Microbial ecology, Metagenomics

## Abstract

Coastal saltmarsh sediments represent an important source of natural methane emissions, much of which originates from quaternary and methylated amines, such as choline and trimethylamine. In this study, we combine DNA stable isotope probing with high throughput sequencing of 16S rRNA genes and ^13^C_2_-choline enriched metagenomes, followed by metagenome data assembly, to identify the key microbes responsible for methanogenesis from choline. Microcosm incubation with ^13^C_2_-choline leads to the formation of trimethylamine and subsequent methane production, suggesting that choline-dependent methanogenesis is a two-step process involving trimethylamine as the key intermediate. Amplicon sequencing analysis identifies *Deltaproteobacteria* of the genera *Pelobacter* as the major choline utilizers. Methanogenic Archaea of the genera *Methanococcoides* become enriched in choline-amended microcosms, indicating their role in methane formation from trimethylamine. The binning of metagenomic DNA results in the identification of bins classified as *Pelobacter* and *Methanococcoides*. Analyses of these bins reveal that *Pelobacter* have the genetic potential to degrade choline to trimethylamine using the choline-trimethylamine lyase pathway, whereas *Methanococcoides* are capable of methanogenesis using the pyrrolysine-containing trimethylamine methyltransferase pathway. Together, our data provide a new insight on the diversity of choline utilizing organisms in coastal sediments and support a syntrophic relationship between Bacteria and Archaea as the dominant route for methanogenesis from choline in this environment.

## Introduction

Coastal saltmarsh sediments represent a highly productive environment, which are predominantly anaerobic and characterized by a high rate of carbon cycling [1]. These sediments represent a significant source of natural methane emissions, resulting from the degradation of organic matter, facilitated by the microorganisms inhabiting these sediments. It has been estimated that between 35 and 90% of the methane production in intertidal mudflats and saltmarshes originates from trimethylamine (TMA) [2, 3]. Quaternary amines are precursors of TMA and are ubiquitous in marine microbes, where they act as osmolytes and essential cellular components. Along with the common marine osmolyte glycine betaine (GBT), choline has been identified as an important precursor of TMA [2, 4]. Choline is a widely distributed component of membrane lipids and is essential for the formation of polar membrane lipids (such as phosphatidylcholine) in all eukaryotes and some bacteria [5]. Standing concentrations of choline have not been assessed in saltmarsh sediments due to the lack of a suitable method, however the concentrations of TMA range from low nanomolar (nM) in oceanic samples to low micromolar (mM) in marine and coastal sediments [6–8]. In marine and coastal systems, cycling of quaternary amines, such as choline, leads to atmospheric fluxes of methylated amines and methane, both of which are important climate-active trace gases [2, 7, 8].

In anaerobic marine and coastal sediments, methanogenesis is a key final step in organic matter degradation. However, the key microbes and the metabolic pathways responsible for methanogenesis from choline are yet to be established. Early studies have focused on the competition between sulphate reducers and methanogens, since sulphate reducers can utilize hydrogen and acetate at much lower concentrations, therefore outcompeting methanogens occupying the same environmental niche [3, 9, 10]. Hence it was thought that methanogens could only thrive in anaerobic sediments depleted of sulphate or utilize alternative non-competitive substrates, such as TMA [2, 4, 11]. Indeed, many methanogens isolated from these sediments, notably *Methanococcoides* of the family *Methanosarcinales* [11, 12], are able to use non-competitive substrates such as TMA, but not acetate, formate nor H_2_/CO_2_ for methanogenesis. These methylotrophic methanogens are known to form strong interactions with bacterial choline degraders [13, 14]. The bacterial choline-to-TMA degradation pathway, through a choline-TMA lyase (encoded by *cutC*), was only elucidated recently and we now know that *cutC* is widely distributed in many marine and coastal sediments [15–17]. It is therefore likely that methanogenesis from choline in anaerobic saltmarsh sediments requires the coupling of the bacterial degradation of choline, with subsequent methanogenesis from TMA. However, whether or not TMA is indeed a key intermediate in choline-dependent methanogenesis in saltmarsh sediments warrants investigation. Indeed, very recently, direct demethylation of choline for methanogenesis has been identified in *Methanococcoides* and *Methanolobus* strains [18–21], although TMA was the preferred substrate over choline and direct demethylation of choline only occurred in the absence of TMA in *Methanococcoides* sp. AM1 [21].

DNA-SIP (stable-isotope probing) is a powerful tool to link microbial identity to metabolic function, through the incorporation of a ^13^C label into the DNA of active microbes, in a culture-independent manner [22]. This technique has recently been applied in several studies to understand biogeochemical cycles in saltmarsh sediments [23–25]. Combining DNA-SIP with shotgun metagenomics and metagenomic binning to retrieve bins from ^13^C-labelled heavy DNA, offers the unique opportunity to uncover the metabolic potentials that are encoded by population genomes from metabolically active microbes [26–28]. In this study, we have used DNA-SIP with ^13^C_2_-labelled choline, followed by amplicon sequencing of 16S rRNA genes, to reveal the active microbial populations responsible for choline degradation. Furthermore, we used metagenomics to retrieve microbial genomes from ^13^C-labelled DNA and demonstrated that methanogenesis from choline in Stiffkey saltmarsh sediments is a two-step process, involving bacterial degradation of choline to TMA by *Pelobacter*, using the choline-TMA lyase pathway, followed by methanogenesis from TMA by the methylotrophic Archaea *Methanococcoides*.

## Materials and methods

### Sampling and microcosm set-ups

Sediment samples were collected from a pond at Stiffkey saltmarsh, Norfolk, UK (latitude 52.96, longitude 00.93) using an acrylic corer on 15/04/2014. The sediment core was transported to the laboratory and stored at 4 °C overnight, until microcosm incubations were set up. The sediment core was then sectioned into discrete layers and the most active, anaerobic 4–6 cm layer was used for analysis.

Microcosm set-up for DNA-SIP experiments were carried out as previously described by Neufeld et al. [29] using ^13^C_2_ choline (the two carbons in the acetyl group of choline was labelled with ^13^C, Sigma-Aldrich). Six sets of triplicate microcosms were used, consisting of 5 g of sediment, 20 ml of sea salts and an initial concentration of 5 mM choline (time point 0; T0). Microcosms were divided equally between ^12^C and ^13^C choline addition (18 each). Choline and TMA concentrations were monitored twice a day. Upon choline depletion one set of triplicate microcosms were sacrificed for ^12^C and ^13^C choline amended microcosms (time point 1, 166.5 h; T1) and an additional 5 mM ^12^C or ^13^C choline was provided to the remaining microcosms. This process of sacrifice and choline addition was repeated (time point 2, 214.5 h; T2). The remaining triplicate microcosms were sacrificed when the third addition of choline was depleted (time point 3, 261 h; T3).

### Ion-exchange chromatography and gas chromatography

The concentrations of choline and TMA were determined twice a day using ion exchange chromatography (IC) on an 881 Compact IC Pro (Metrohm, Herisau, Switzerland) as described previously [30]. 100 μl aliquots of liquid medium from each microcosm was filter-sterilized with a 0.2 μm pore size centrifuge filter and diluted tenfold in MillQ water prior to IC-analysis.

Methane concentration in the head-space of the microcosms was monitored daily. Gas chromatography (GC) was carried out to quantify methane, using an Agilent 6890 FID instrument with a Porapak Q column with N_2_ carrier gas flowing at 20 ml min^−1^. The temperature set up was as follows: injector 150 °C, column 125 °C and detector 200 °C. An injection volume of 100 µl was used for all measurements. Methane concentrations were determined by peak area against a set of standards of known concentrations covering the measured range.

### DNA extraction and gradient fractionation

DNA was extracted from ~500 mg of sample from the sacrificed microcosms, using the Fast DNA soil extraction kit (MP Bioscience, UK) according to the manufacturer’s instructions. DNA was extracted from the unamended sediment (T0), and the sacrificed microcosm sediments obtained at 166.5 h (T1), at 214.5 h (T2) and at 261 h after choline addition (T3). DNA concentrations were estimated using a spectrophotometer (NanoDrop ND-1000) and found to be in a range of 150–200 ng/μl. Aliquots of DNA (3 μg of DNA, 15–20 μl) were subjected to ultracentrifugation in CsCl. After centrifugation, between 12 and 13 CsCl fractions were collected by piercing the top and the bottom of the tube using a 23-Gauge needle. The density of each fraction was measured using a digital refractometer and DNA was recovered using polyethylene glycol as described previously [29].

### Amplicon sequencing of 16S rRNA genes

Microbial community analyses of the 16S rRNA gene amplicons were performed using unfractionated DNA from T0, T1, T2 and T3 and DNA extracted from SIP gradient heavy and light fractions. The primers used for amplifying the 16S rRNA genes were designed to amplify both Bacteria and Archaea [31]. Amplicon sequencing of the 16S rRNA gene was carried on an Illumina Miseq platform, as described by Caporaso et al. [31], on the fractionated T0 ‘heavy’ and ‘light’ fractions, and for T1, T2 and T3, both ^12^C and ^13^C, heavy and light fractions. As a control against amplification of DNA originating from the laboratory and the DNA extraction kit, we ran nuclease-free water through the soil DNA extraction kit and Illumina PCR steps, referred to as the negative control. Multiplex 515F/806R paired-end 16S rRNA bacterial and archaeal community sequencing primers described by Caporaso et al. [31] were used.

16S rRNA gene amplicon reads were joined, de-multiplexed, trimmed and filtered. Singletons and chimeras were removed, reads were normalized and Operational Taxonomic Unit (OTU) binning was performed using the open-source bioinformatics pipeline Quantitative Insights Into Microbial Ecology (QIIME), MacQIIME version 1.8.0 [31]. Sequences were joined using fastq-join [32], singletons were removed, then the sequences were trimmed (240 bp) and quality filtered (maximum expected error threshold of 0.5) using USEARCH [33]. UCHIME was used for chimera detection and removal, with the Broad Microbiome Utilities ‘Gold.fa’ reference database [34]. For rarefaction analysis, approximately-maximum-likelihood phylogenetic trees were constructed using FastTree 2.1.3 [35]. OTU binning was performed with the UCLUST method with a cut off of 97% sequence identity using the RDP Classifier 2.2 against the Greengenes 13_8 dataset [33, 36, 37]. The QIIME processing yielded sequences of 240 bp, rarefied to a sequencing depth of 36,372 reads per sample (normalized to the smallest sample).

### Metagenome sequencing and binning

Metagenomic sequencing was carried out on triplicate unfractionated time-point 0 and triplicate biological replicates of fractionated ^13^C-labelled time-point 3 DNA from both ‘heavy’ and ‘light’ fractions. The metagenomic libraries were constructed using the NEBNext Ultra DNA Library Prep Kit for Illumina (NEB, Hitchin, UK) and NEBNext Multiplex Oligos for Illumina (Index Primers Set 1), then sequenced using the MiSeq Reagent Kit v3 with 600 cycles.

The removal of adapters and quality trimming was carried out using sickle (quality > 20 average per kmer; [38]). Reads were assembled into contigs using Megahit v1.1.2, assigned to bins using Metabat v0.32.5 and annotated with Prokka [39–42]. Reads were mapped against contigs using BWA-MEM and resultant SAM and BAM files manipulated with Samtools v1.6 [43, 44]. To validate the completeness of each bin (assuming each bin represents a single organism), the percentage of the 40 core prokaryotic Clusters of Orthologous Groups of proteins (COGs) identified within each bin was calculated, based on previous approaches [45–47]. Estimation of contamination in each bin was carried out using the CheckM program [48]. Taxonomy was first assigned for each bin using specI [49]. Secondly, for the bins lacking a specI taxonomic assignment, blastp was carried out using the core COGs against the NCBI Nucleotide collection database (August 2017). Finally, for the remaining bins with too few core genes identified, blastp of all the contigs was carried out against the NCBI Nucleotide collection (nt) with an Hsp *e*-value cut-off of 1e–30.

Read data have been submitted to the European Nucleotide Archive (ENA) under the study accession number PRJEB23843.

### Statistical and bioinformatics analysis

PRIMER v.6.8 (PRIMER-E, Plymouth, UK) was used for statistical analyses of the taxonomic data [50, 51]. The patterns of relative abundance for OTUs (16S rRNA gene amplicons) or bins (metagenome) from each sample were ordinated to each other using non-metric multidimensional scaling (MDS) analysis, allowing the comparison of compositional similarity between samples. Resemblance matrices were calculated on untransformed standardized OTU/bin relative abundance data using Bray–Curtis similarity analysis. MDS analysis was applied to the matrices using the default settings in PRIMER, with Kruskal’s stress formula 1, a minimum stress of 0.01 and 50 restarts. Similarity percentage analysis (SIMPER) was used to determine the percentage of similarity and dissimilarity between sets of microbial communities; i.e. time-points, ^12^C and ^13^C samples and fractions. SIMPER was applied to the resemblance matrices in PRIMER using the default settings.

To understand the relative prevalence of selected functional metabolic pathways in the metagenome data, BLAST analysis of both the binned and the un-binned metagenome data was carried out. The representative protein sequence queries were selected because they had proven role in choline and TMA degradation. Additionally, a control query for GBT degradation was selected. The functional metabolic genes used were; *cutC* encoding choline-TMA lyase [16, 52], *mttB* encoding the pyrrolysine-containing TMA methyltransferase [53], and the non-pyrrolysine containing GBT: corrinoid methyltransferase, *mtgB* [19]. After BLAST analysis, the resultant hits were aligned with MUSCLE 3.5 [54] and maximum likelihood phylogenetic trees were reconstructed using PhyML 3.0 [55] with default settings (i.e. HKY85 model of nucleotide substitutions, ts/tv ratio = 4.00, BioNJ starting tree). This phylogenetic analysis predicted true-positive hits, based on their clustering with representative, validated functional genes, whilst false-positive hits (those that clustered closest to genes with alternative functions) were rejected. The positive BLAST hits were normalized to gene length and sample size.

## Results

### Methanogenesis from choline involved TMA as a key intermediate in saltmarsh sediments

**Fig. 1:**
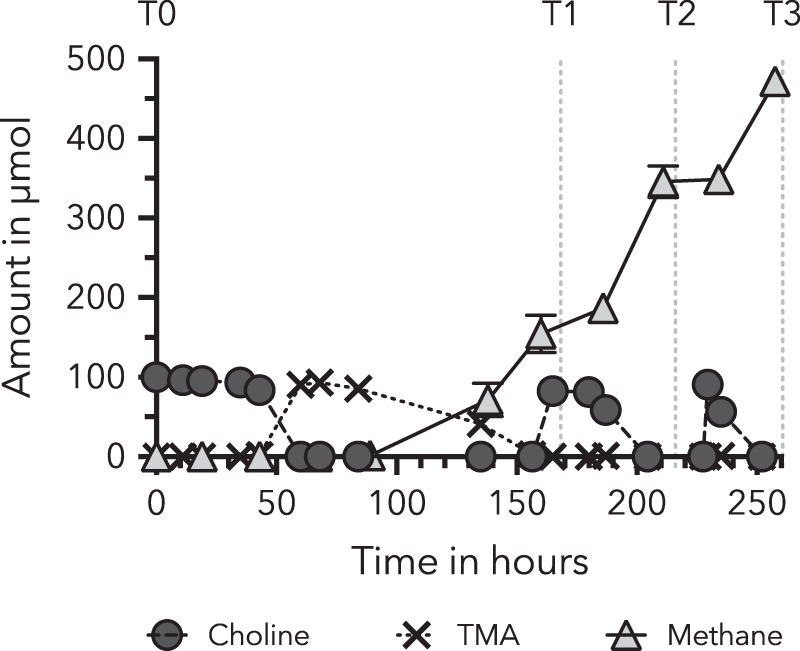
Choline, trimethylamine (TMA) and methane dynamics during the microcosm incubations. Choline and TMA were determined by ion-exchange chromatography; methane was determined by gas chromatography. Time-points at which the microcosms were sampled are indicated by the labels T0, T1 (166.5 h), T2 (214.5 h) and T3 (261 h). Error bars represent standard deviations from six to nine biological replicates

We have hypothesized that methanogenesis from choline in saltmarsh sediments is a two-step process, involving the bacterial degradation of choline to TMA and subsequent methane formation from TMA by methylotrophic methanogens. Triplicate choline enrichment microcosms (with ^13^C_2_-labelled choline and unlabelled choline), were monitored for choline and its metabolites using IC and methane formation was quantified by GC. The IC data presented in Fig. 1 showed that the addition of choline (100 μmol) at T0 was depleted and an equivalent amount of TMA was produced after 60 h. The released TMA was fully degraded after a further 7 days (186 h) and approximately two times the amount of methane was produced (202 μmol). The time taken to degrade all the choline decreased with the two subsequent choline additions, which were 48 and 25 h respectively (Fig. 1). TMA was not detectable after the second and third choline additions, suggesting a rapid consumption by the microbial community (Fig. 1). Following the second choline addition, the methane production was roughly equal to the choline degradation rate (Fig. 1). The cumulative amount of choline after the three additions was 300 μmol (per incubation) and the average amount of methane produced was 469 μmol (per incubation).

### Microbial community analyses of the 16S rRNA genes from the choline-amended microcosms and the SIP gradient fractions by amplicon sequencing

To uncover the microbial community response to choline amendment and to uncover the microbes involved in choline transformation, we sequenced the 16S rRNA genes from both the light and heavy fractions of the DNA-SIP microcosms over the time course experiment. Sequencing using the Illumina Miseq platform yielded 4,806,794 reads. OTUs were assigned to 3,353,870 sequences, with an average sequencing depth of 74,869 reads per sample. The QIIME processing yielded sequences of 240 bp, rarefied to the lowest sequencing depth of 36,372 reads per sample. Sequencing reads were assigned to 940 OTUs at 97% sequence identity cut-offs.

**Fig. 2:**
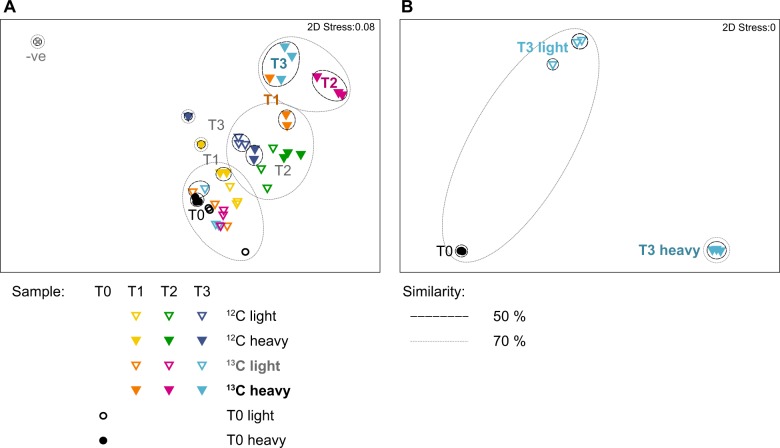
Multidimensional scaling (MDS) plots of fractionated choline DNA-SIP samples taken before enrichment (T0) and at time-points 1 (T1), 2 (T2) and 3 (T3) incubated with either ^12^C-choline (^12^C light and ^12^C heavy) or ^13^C_2_-choline (^13^C light and ^13^C heavy). **a** 16S rRNA gene amplicon data based on rarefied absolute abundance of OTUs (97% cut-off), with an additional blank negative control (−ve) sample. **b** Metagenome bins abundance, normalized to contig length and sample size

Over the SIP time course there was a progressive community shift from T0 through T1 to T2 and T3. MDS analysis showed that T2 and T3 heavy fractions of the ^13^C_2_-choline microcosms were significantly different from the unlabelled microcosms, an indication that the labelling was successful for the enriched ^13^C_2_-choline metabolizing bacteria (Fig. 2a). The light fractions from ^13^C_2_-microcosms clustered closely with T0 samples (<25% dissimilar; Fig. 2a). The negative control was most dissimilar to all other samples (92.9–99.8%), and clustered separately from all other samples in the MDS plot (Table 1, Fig. 2a).

**Table 1:** Dissimilarity matrix of the SIMPER analysis showing average dissimilarity between triplicate samples for 16 S rRNA gene OTUs and metagenome bins for each time point (T0, T1, T2 and T3) and light versus heavy fractions

**Fig. 3:**
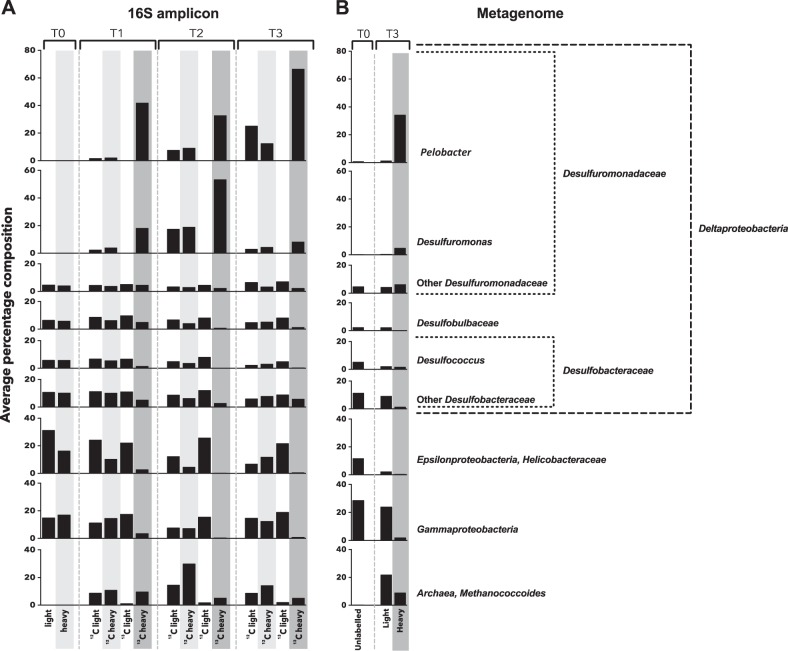
Relative abundance of the top 9 taxonomically assignments as identified by similarity percentage analysis (SIMPER). **a** 16S rRNA gene amplicon OTUs. **b** Metagenome bins

The SIMPER analysis for the T1 the heavy and light ^13^C_2_-choline fractions showed these two communities had 79% dissimilarity, which increased to 96% for T2 and 93% for T3 (Table 1), suggesting a clear separation of an active microbial population from the community in response to ^13^C_2_-choline amendment. Yet the unlabelled ^12^C-choline microcosms showed <40% dissimilarity between the heavy and light fractions (Table 1). To determine the key OTUs responsible for the observed dissimilarity of the microbial communities between the heavy and light fractions, SIMPER analysis was applied. Data presented in Fig. 3 indicate that there was an increase in the Archaea *Methanococcoides*, and *Deltaproteobacteria* (*Desulfuromonas* and *Pelobacter)*, whilst other taxa such as *Epsilonproteobacteria (Helicobacteraceae)* and *Gammaproteobacteria* declined in relative abundance after choline additions (Fig. 3). These same taxonomic groups (i.e. *Pelobacter* and *Methanococcoides*) also showed enrichment when much lower levels of choline (150 μM, 1.5 mM) were added to microcosms using the Stiffkey saltmarsh sediments (Fig. S1).

**Fig. 4:**
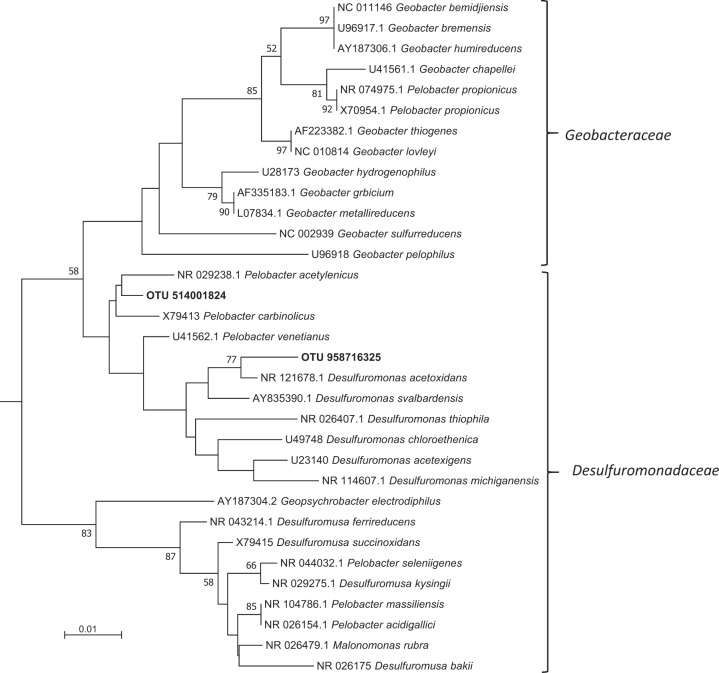
Neighbour-joining phylogenetic tree of 16S rRNA genes of *Desulfuromonadaceae*. Sequence alignment was carried out using the ClustalW program based on partial 16S rRNA genes (1320 bp) from representative species from the *Geobacteraceae* and *Desulfuromonadaceae* families and the two most prevalent OTUs derived from amplicon sequencing of 16S rRNA genes from the choline DNA-SIP heavy fractions (OTU 514001824 and OTU 958716325). *Sedimentation selenatireducens* and *Desulfoluna spongiiphila* were used as the outgroup to root the tree. The percentage of replicate trees in which the associated taxa clustered together in the bootstrap test (500 replicates) are shown next to the branches. Only bootstrap values greater than 50% are shown. The evolutionary distances were computed using the Maximum Composite Likelihood method in the MEGA7 package [62]. The scale bar indicates evolutionary distance in mutations per residue

Two *Deltaproteobacteria* OTUs of the *Desulfuromonadaceae* family (OTU 514001824 and OTU 958716325), classified as *Pelobacter* and *Desulfuromonas* respectively (Fig. 4), were significantly enriched in the ^13^C-choline heavy fractions which, together, accounted for >80% reads in ^13^C-heavy fractions (Table S1). The SIMPER analysis indicated that these two taxonomic groups, *Pelobacter* and *Desulfuromonas* OTUs, accounted for 19% and 12%, respectively, of community shift between all fractions and time points (Table S3). These OTUs accounted for <0.1% of the sequences in T0 samples before ^13^C_2_-choline enrichment (Fig. 3). *Methanococcoides* was enriched in all fractions, i.e. ‘heavy’ and ‘light’ for both ^12^C and ^13^C-choline microcosms (Table S1).

### Metagenome sequencing of heavy isotope labelled DNA from ^13^C_2_-choline DNA-SIP and bins

To gain a better understanding of the metabolic potential in these enriched taxa (i.e. *Pelobacter*), we sequenced triplicate ^13^C light and triplicate ^13^C heavy fractions of time point 3 (T3) DNA-SIP fractions, together with the triplicate unfractionated T0 samples. The dataset was sequenced using an Illumina MiSeq with 2 × 300 paired-end cycles, resulting in ~186 Mb sequencing data from 11 runs. After quality trimming and filtering, ~116 Mb were mapped to contigs. For the T0, T3 light and T3 heavy samples the percentage of reads that mapped to contigs accounted for 49%, 65% and 80% of total reads, respectively. These resulted in 230,960 contigs, with a minimum length cut-off of 1 kb, which were assigned to 270 bins (Table S2).

The MDS analysis of the metagenome bins showed that the T0 and T3 heavy fraction samples clustered closely with themselves, with >70% similarity (Fig. 2B). The T3 light fraction samples showed lower similarity. The T0, T3 light and T3 heavy clusters were distinct from each other. The MDS analysis of metagenome bins therefore agrees with the results from amplicon sequencing data on 16S rRNA genes in that significant enrichment of active microbes involved in choline metabolism had occurred in the SIP microcosms.

SIMPER analyses revealed that the T3 ^13^C-choline heavy labelled samples were highly dissimilar to both the T0 (87%) and the T3 light samples (80%; Table 1). In agreement with the 16S rRNA gene amplicon data (Table S3), the SIMPER analyses of the metagenome revealed an increase in the Archaea *Methanococcoides* between the T0 samples and T3 light fraction samples and confirmed enrichment of *Pelobacter* in the T3 heavy fraction samples (Table S4).

The taxonomy assigned to the 270 metagenome bins was determined to the greatest possible resolution, which varied between class and species level (Table S2). The most prevalent taxonomic assignment among the 270 bins (irrespective of relative abundance) was *Pelobacter* (40 bins), *Desulfovibrio* (19 bins) and *Methanococcoides* (13 bins; Table S2).

### Functional gene profiling of the metagenomes and microbial genomes retrieved from DNA-SIP

**Fig. 5:**
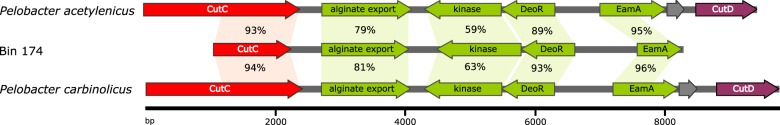
Alignment of the *cutC*-homologue containing contig from the ^13^C choline enriched bin 174 with the closest genome matches—*Pelobacter acetylenicus* and *Pelobacter carbinolicus*

Although each of the metagenome bins only represents a partial genome of the assigned species, they provided a valuable source for mining the functional genetic potential involved in choline degradation and methane formation. Specifically, these bins were screened for the presence of the metabolic genes involved in converting choline to TMA (*cutC*) and TMA to methane (*mttB*). Data presented in Table 2 and Table S2 revealed that *cutC* was indeed present in bins that were assigned to *Pelobacter* (e.g. 5, 134, 174, 227 and 252). Similarly, the *mttB* gene was also found in bins that were assigned to *Methanococcoides* (e.g. 68, 76, 117 and 267). Additionally, a complete set of proteins (*mtaA*, *mtaB* and *mtaC*) required for methanogenesis from methanol are also found in these *Methanococcoides* bins (e.g. 68 and 267). Analysis of the recovered contigs containing these key functional genes showed that for Bin 174 the *cutC* containing contig showed high gene synteny as well as sequence similarity to *Pelobacter* isolates (Fig. 5).

**Table 2:** Selected bins containing homologues of functional genes (*cutC* and *mttB*) involved in choline-dependent methanogenesis

Bin no.	Taxonomy	Length (bp)^a^	Genome completeness	Top BLASTp hit of CutC or MttB and identity (%)			Relative abundance		
							T0	T3 light	T3 heavy
174	*Pelobacter cabinolicus* [30S ribosomal protein S5, 96%]	2,471,953	48%	*cutC*	*Pelobacter carbinolicus*	93	0.02	0.01	0.29
134	*Pelobacter cabinolicus* [30S ribosomal protein S2, 80%]	2,898,805	15%	*cutC*	*Desulfobacteraceae*	70	0.01	0.03	0.91
105	*Desulfobacter curvatus* [30S ribosomal protein S12, 99%]	1,645,370	68%	*cutC*	*Desulfoluna spongiiphila*	80	0.02	0.03	0.75
162	*Desulfobacter postgatei* [50S ribosomal protein L14, 98%]	1,431,515	30%	*cutC*	*Sporomusa silvacetica*	72	0.01	0.03	0.56
14	*Desulfobacter vibrioformis* [50S ribosomal protein L16, 96%]	3,502,099	15%	*cutC*	*Desulfosporosinus acidiphilus*	79	0.01	0.13	0.82
145	*Desulfovibrio salexigens* [30S ribosomal protein S2, 98%]	3,824,891	40%	*cutC*	*Desulfoluna spongiiphila*	79	0.02	0.03	0.6
260	*Desulfuromonas acetoxidans* [30S ribosomal protein S2, 72%]	1,754,599	13%	*cutC*	*Desulfoluna spongiiphila*	74	0.03	0.03	0.36
68	*Methanococcoides burtonii* [50S ribosomal protein L1, 96%]	1,493,633	28%	*mttB*	*Methanococcoides vulcani*	71	0.01	1.35	0.54
267	*Methanococcoides burtonii* [50S ribosomal protein L1, 91%]	1,836,990	38%	*mttB*	*Methanosarcina*	52	0.02	0.9	0.64

^a^Only show bins of length >1 millon bp

**Fig. 6:**
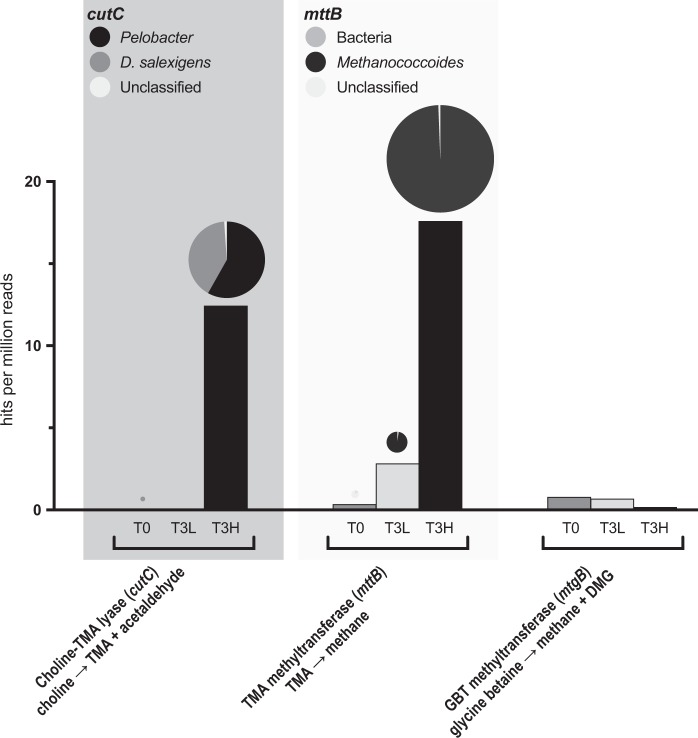
Analyses of functional gene abundance in the un-binned metagenome datasets obtained from saltmarsh sediments before (T0) and after choline DNA-SIP (L-, light and H-, heavy fractions of T3). The *cutC* gene encodes a choline-TMA lyase ([17, 16]). *mttB* encodes a pyrrolysine-containing TMA methyltransferase [63, 64] and *mtgB* encodes a non-pyrrolysine glycine betaine (GBT) methyltransferase responsible for direct demethylation of GBT to dimethylglycine (DMG) and methane [19]. Relative abundance was achieved by normalizing to the length and abundance of the *recA* gene. Note that the normalized *mttB* counts are not exclusive to methanogens and a complete separation of bacterial pyrrolysine-containing *mttB* from their archaeal counterparts through phylogeny analysis was not possible [64]. The pie charts give a breakdown of the phylogeny of the hits against a reference tree for *cutC* or *mttB*, and the size of the pie charts reflects relative abundance of sequences retrieved from the metagenomes. The *cutC* sequences grouped with either *Pelobacter* (*P. acetylenicus* and *P. carbinolicus*) or *Desulfovibrio salexigens* whereas the archaeal *mttB* sequences clustered within *Methanococcoides*

BLAST analysis of the key genes in the functional metabolic pathways against the unassembled metagenome reads revealed an increase in the two expected pathways involved in methanogenesis from choline (Fig. 6). The *cutC* gene was detected at very low levels (<0.05 hits per million reads) in both the T0 and T3 light metagenome samples, but we detected 12.4 hits per million reads in the T3 heavy samples. Phylogenetic analysis of the *cutC* genes extracted from the T3 heavy metagenome samples showed that the majority (~60%) of the *cutC* sequences originated from *Pelobacter*. The *mttB* gene was also detected at low levels (<0.4 hits per million reads) in the T0 samples, then increased to 2.8 in T3 light and 17.6 hits per million reads in T3 heavy samples. Phylogenetic analyses of *mttB* sequences retrieved from these metagenomes confirmed that they were from *Methanococcoides*. Interestingly, our analyses of the nonpyrrolysine-containing GBT methyltransferase *mtgB* gene, which is responsible for the direct demethylation of GBT [19], showed little variation in abundance (0.2–0.8 hits per million reads) between T0 and T3 samples.

## Discussion

Coastal saltmarshes represent a significant natural source of methane in the global methane budget. Our understanding of the key microbes and metabolic pathways responsible for methanogenesis by microbial populations inhabiting these sediments is still very limited. In this study, we have shown that the *Deltaproteobacteria* genera *Pelobacter* and the Archaea, *Methanococcoides* were the predominant species involved in methanogenesis from Stiffkey saltmarsh sediments. These microbes were identified through a combination of DNA-SIP, high-throughput sequencing analyses of the 16S rRNA genes, and ^13^C-enriched metagenomics, on which binning and subsequent reconstruction of the metagenome bins was performed.

DNA-SIP and amplicon sequencing analyses clearly showed that the *Desulfuromonadaceae*, particularly *Pelobacter* and *Desulfuromonas* were virtually exclusively found in the ^13^C heavy fractions, suggesting that these obligate anaerobes incorporated heavy carbon from the ^13^C_2_-choline into their DNA. Indeed, cultivated representatives of *Pelobacter* have previously been isolated from various marine and coastal sediments and can grow on choline while producing TMA [56, 57]. This was further supported by subsequent analyses of the retrieved population genomes that were assigned to *Pelobacter* (5, 134, 174, 227 and 252), showing the presence of *cutC* and associated genes involved in the choline-TMA lyase pathway (Table 2, Fig. 5). *Desulfuromonas* isolates such as *Desulfuromonas acetoxidans* [58] and *Desulfuromonas svalbardensis* [59], on the other hand, are not known to grow on choline and no *cutC* homologues were found in the genomes of the two aforementioned strains. Yet, our data presented in Fig. 3 appears to support the role of as-yet uncultivated novel *Desulfuromonas* strains in these sediments in choline degradation. However, it is also likely that *Desulfuromonas* was labelled through cross-feeding of ^13^C-acetate released by *Pelobacter*. Indeed, both *Pelobacter carbinolicus* and *Pelobacter acetylenicus* constitutively expressed an acetaldehyde dehydrogenase, converting acetaldehyde to acetate in pure culture or co-culture with a methanogen [60]. Interestingly, a significant proportion of *cutC* sequences retrieved from the ^13^C-heavy metagenomes are classified as *Desulfovibrio salexigens* (Fig. 6). Furthermore, several metagenome-assembled bins related to this sulphate-reducing bacterium were also found although the SpecI matches of these bins were <90% identical to those of *Desulfovibrio salexigens* (Table S2). However, the 16S rRNA gene amplicon sequencing indicated that *Desulfovibrio* (Family *Desulfovibrionaceae*) was not abundant (<1%) in this saltmarsh sediment and our SIMPER analyses did not support their role in contributing to community shift during choline SIP incubations (Fig. 3, Table S1). Clearly, the role of *Desulfuromonas* and *Desulfovibrio* in choline degradation warrants further investigation.

Amplicon sequencing of 16S rRNA genes showed that *Methanococcoides* were also enriched over time, however, they showed no preferential enrichment in the heavy ^13^C fractions compared to either the light fractions or the heavy fractions from the ^12^C-choline control microcosms (Fig. 3). *Methanococcoides* cannot use acetate and therefore could not incorporate ^13^C label from the acetyl group of ^13^C_2_-choline [14]. They can, however, use TMA for methanogenesis [14]. TMA, a metabolite of bacterial choline degradation [16, 17], was indeed found in our choline-amended microcosms (Fig. 1). We therefore postulate that the enrichment of *Methanococcoides* in these microcosms was due to their growth on TMA generated by bacterial choline degradation. To further support our hypothesis, our analyses of the population genomes retrieved from metagenomics sequences showed the presence of *mttB*, TMA-methyltransferase, a key gene involved in methanogenesis from TMA, in *Methanococcoides* bins (Table 2).

**Fig. 7:**
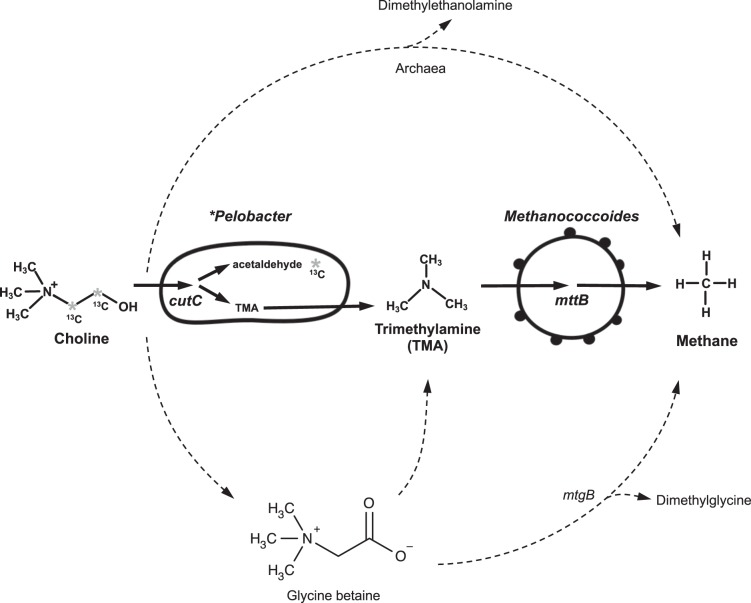
Currently known pathways for methanogenesis from choline. Choline can be converted to trimethylamine (TMA) by either a bacterial choline-TMA lyase (encoded by *cutC* [17, 16]) or indirectly through glycine betaine (GBT) as the intermediate. Methanogenesis from TMA by methanogenic Archaea requires the key enzyme, pyrrolysine-containing TMA methyltransferase encoded by *mttB* [53]. Direct demethylation of choline and GBT can also support methanogenesis and the GBT methyltransferase (*mtgB*) has been identified very recently [19, 21] whereas genes responsible for direct choline demethylation to methane have not yet been identified [20, 21]

Metagenomic sequencing of the ^13^C-enriched DNA and binning of the metagenomic data not only provided a better understanding of the metabolic potential of the key functional microbes identified by SIP, *Desulfuromonadaceae* (e.g. *Pelobacter*) and *Methanococcoides*, but also provided an opportunity to elucidate major pathway for methanogenesis from choline in the sediments. There are several possible routes for choline transformation to methane in anaerobic sediments (Fig. 7): (1) direct demethylation of choline to methane (the enzyme responsible has yet to be characterized [11, 21]); (2) TMA formation from choline followed by methanogenesis from TMA, or (3) demethylation of GBT to methane. The occurrence of TMA during the anaerobic degradation of choline in our microcosms (Fig. 1) suggests that direct choline demethylation was not the dominant route for methanogenesis. Instead, our data suggest that choline was initially converted to TMA, which then served as the substrate for methanogenesis. TMA formation from choline can be achieved through either a choline-TMA lyase encoded by *cutC* or, alternatively, GBT as the intermediate (Fig. 7). We, however, did not observe any significant accumulation of GBT in the microcosms although the method is capable of quantifying GBT, TMA and choline simultaneously [61]. To further support our hypothesis, we compared the relative abundance of key metabolic genes in the un-amended salt-marsh sediment and after choline enrichment. The relative capacity of the microbial community to degrade choline to TMA and subsequently TMA to methane markedly increased after SIP incubations, as evidenced by a substantial increase in relative abundance of both *cutC* (choline to TMA) and *mttB* (TMA to methane) (Fig. 6). In contrast, the relative abundance of *mtgB*, encoding the GBT methyltransferase responsible for direct demethylation of GBT [19], was more abundant before enrichment and showed no response to choline amendment in microcosms, suggesting that GBT was not a major intermediate in methanogenesis from choline.

Using a combination of DNA-SIP with ^13^C_2_-labelled choline, 16S rRNA gene sequencing and metagenome sequencing we have identified *Deltaproteobacteria*, of the genera *Pelobacter* as the major choline-utilizers and TMA producers, whilst the methanogenic Archaea *Methanococcoides* was also enriched and involved in methane formation from TMA. Metagenome and metabolite data showed a significant enrichment in the choline degradation pathway via TMA to methane and a correlating intermediate release of TMA and a final accumulation of methane in the choline enrichment microcosms. This all indicated that a syntrophic relationship between Bacteria and Archaea was the dominant route for methanogenesis from choline in Stiffkey saltmarsh sediments.

## Electronic supplementary material

supplementary information

supplementary table 1

supplementary table 2

supplementary table 3

supplementary table 4

Supplementary Figure
