# Supplementary material for: Deltaproteobacteria (Pelobacter) and Methanococcoides are responsible for choline-dependent methanogenesis in a coastal saltmarsh sediment
Source: ISME J. 2018 Sep 11;13(2):277–89. doi: 10.1038/s41396-018-0269-8 (PMC6331629; doi:10.1038/s41396-018-0269-8)
Supplement: Supplementary file 1 — supplementary information [file 41396_2018_269_MOESM1_ESM.docx]

**Supplementary information**

**Table S1** Relative abundance of top 50 OTUs of 16S rRNA genes for all time-points.

**Table S2** Taxonomic assignment and relative abundance of all 270 metagenome bins in time-points 0 (unfractionated DNA) and time-point 3 (light and heavy fractions). Also included are number of contigs assembled for each bin, combined contig length in bp and COG gene counts of 40 single-copied core COGs.

**Table S3** SIMPER analysis of the 16S rRNA gene taxonomy data, comparison of all time-points. The 20 OTUs contributing greatest to community changes are shown.

**Table S4** SIMPER analysis of the metagenome bin data, comparison of time-points 0, unfractionated DNA and time-point 3, representative light and heavy fractions. The 20 taxonomic groups contributing greatest to community changes are shown.

**Figure S1.** Relative abundance of the top 9 taxonomically assignments as identified by similarity percentage analysis (SIMPER) for 16S rRNA gene amplicon OTUs at varying concentrations of choline. Microcosms were set up in three replicates using sediments taken from Stiffkey saltmarsh on 09/05/2016 and choline was added to a final concentration of 150 μM, 1.5 mM and 150 mM respectively. Amplicon sequencing of 16S rRNA genes was performed using DNA extracted from T1 (167 hr), T2 (215 hr) and T3 (261 hr), respectively.
