# Supplementary material for: Deltaproteobacteria (Pelobacter) and Methanococcoides are responsible for choline-dependent methanogenesis in a coastal saltmarsh sediment
Source: ISME J. 2018 Sep 11;13(2):277–89. doi: 10.1038/s41396-018-0269-8 (PMC6331629; doi:10.1038/s41396-018-0269-8)
Supplement: Supplementary file 5 — supplementary table 4 [file 41396_2018_269_MOESM5_ESM.docx]

**Table S4** SIMPER analysis of the metagenome Bin data, comparison of time-points 0 and time-point 3 (light and heavy fractions) of ^13^C_2_-choline SIP fractions. Showing the top 20 greatest contributing taxonomies to community changes.

|  |  |  |  |  | Relative abundance | | |
| --- | --- | --- | --- | --- | --- | --- | --- |
| Bin | Closest taxonomical hit | Av. dissim | Contrib. % | Cumulative % | T0 | T3 light | T3 heavy |
| 1 | *Euryarchaeota, Methanomicrobia, Methanosarcinales, Methanosarcinaceae, Methanococcoides burtonii* | 2.928 | 4.132 | 4.13 | 0.02 | 9.35 | 1.33 |
| 3 | Virus | 1.148 | 1.621 | 5.75 | 0.02 | 3.53 | 0.867 |
| 33 | Virus | 0.8411 | 1.187 | 6.94 | 0.03 | 2.34 | 0.52 |
| 4 | *Deltaproteobacteria, Desulfobacterales, Desulfobacteraceae, Desulfobacter* | 0.6714 | 0.9476 | 7.89 | 0.01 | 0.0333 | 2.08 |
| 117 | *Euryarchaeota, Methanomicrobia, Methanosarcinales, Methanosarcinaceae, Methanococcoides methylutens* MM2 | 0.6267 | 0.8846 | 8.77 | 0.01 | 1.98 | 0.537 |
| 238 | *Euryarchaeota, Methanomicrobia, Methanosarcinales, Methanosarcinaceae, Methanococcoides burtonii* DSM 6242 | 0.5311 | 0.7496 | 9.52 | 0.01 | 1.69 | 0.2 |
| 114 | *Euryarchaeota, Methanomicrobia, Methanosarcinales, Methanosarcinaceae, Methanococcoides burtonii* | 0.5055 | 0.7135 | 10.24 | 0.01 | 1.6 | 0.513 |
| 64 | *Euryarchaeota, Methanomicrobia, Methanosarcinales, Methanosarcinaceae, Methanococcoides burtonii* | 0.4655 | 0.657 | 10.89 | 0.01 | 1.48 | 0.243 |
| 68 | *Euryarchaeota, Methanomicrobia, Methanosarcinales, Methanosarcinaceae, Methanococcoides burtonii* | 0.4319 | 0.6096 | 11.50 | 0.01 | 1.36 | 0.543 |
| 5 | *Deltaproteobacteria, Desulfuromonadales, Desulfuromonadaceae, Pelobacteraceae, Pelobacter* | 0.4156 | 0.5865 | 12.09 | 0.01 | 0.0167 | 1.29 |
| 10 | *Deltaproteobacteria, Desulfobacterales, Desulfobacteraceae, Desulfobacter* | 0.4092 | 0.5776 | 12.67 | 0.01 | 0.03 | 1.27 |
| 7 | *Deltaproteobacteria, Desulfuromonadales, Desulfuromonadaceae, Pelobacteraceae* | 0.4025 | 0.568 | 13.23 | 0.01 | 0.03 | 1.25 |
| 28 | *Gammaproteobacteria* | 0.3988 | 0.5628 | 13.80 | 0.983 | 1.09 | 0.03 |
| 71 | *Deltaproteobacteria, Desulfuromonadales, Desulfuromonadaceae, Pelobacteraceae, Pelobacter* | 0.3983 | 0.5622 | 14.36 | 0.01 | 0.0133 | 1.24 |
| 8 | *Deltaproteobacteria, Desulfuromonadales, Desulfuromonadaceae, Pelobacteraceae, Pelobacter* | 0.3973 | 0.5608 | 14.92 | 0.01 | 0.0233 | 1.24 |
| 180 | *Proteobacteria, Epsilonproteobacteria, Campylobacterales, Helicobacteraceae, Sulfurovum* sp. NBC37-1 | 0.3961 | 0.5591 | 15.48 | 1.25 | 0.23 | 0.0267 |
| 89 | Virus | 0.3865 | 0.5455 | 16.02 | 0.04 | 1.04 | 0.423 |
| 122 | *Deltaproteobacteria, Desulfuromonadales, Desulfuromonadaceae, Pelobacteraceae, Pelobacter cabinolicus* | 0.3845 | 0.5427 | 16.57 | 0.0167 | 0.0133 | 1.2 |
| 37 | *Deltaproteobacteria, Desulfobacterales* | 0.3793 | 0.5353 | 17.10 | 0.85 | 1.04 | 0.0267 |
| 154 | *Deltaproteobacteria, Desulfuromonadales, Desulfuromonadaceae, Pelobacteraceae, Pelobacter* | 0.3756 | 0.5302 | 17.63 | 0.01 | 0.0633 | 1.17 |
