# Supplementary figures and images for: Deltaproteobacteria (Pelobacter) and Methanococcoides are responsible for choline-dependent methanogenesis in a coastal saltmarsh sediment
Source: ISME J. 2018 Sep 11;13(2):277–89. doi: 10.1038/s41396-018-0269-8 (PMC6331629; doi:10.1038/s41396-018-0269-8)

# Choline concentration

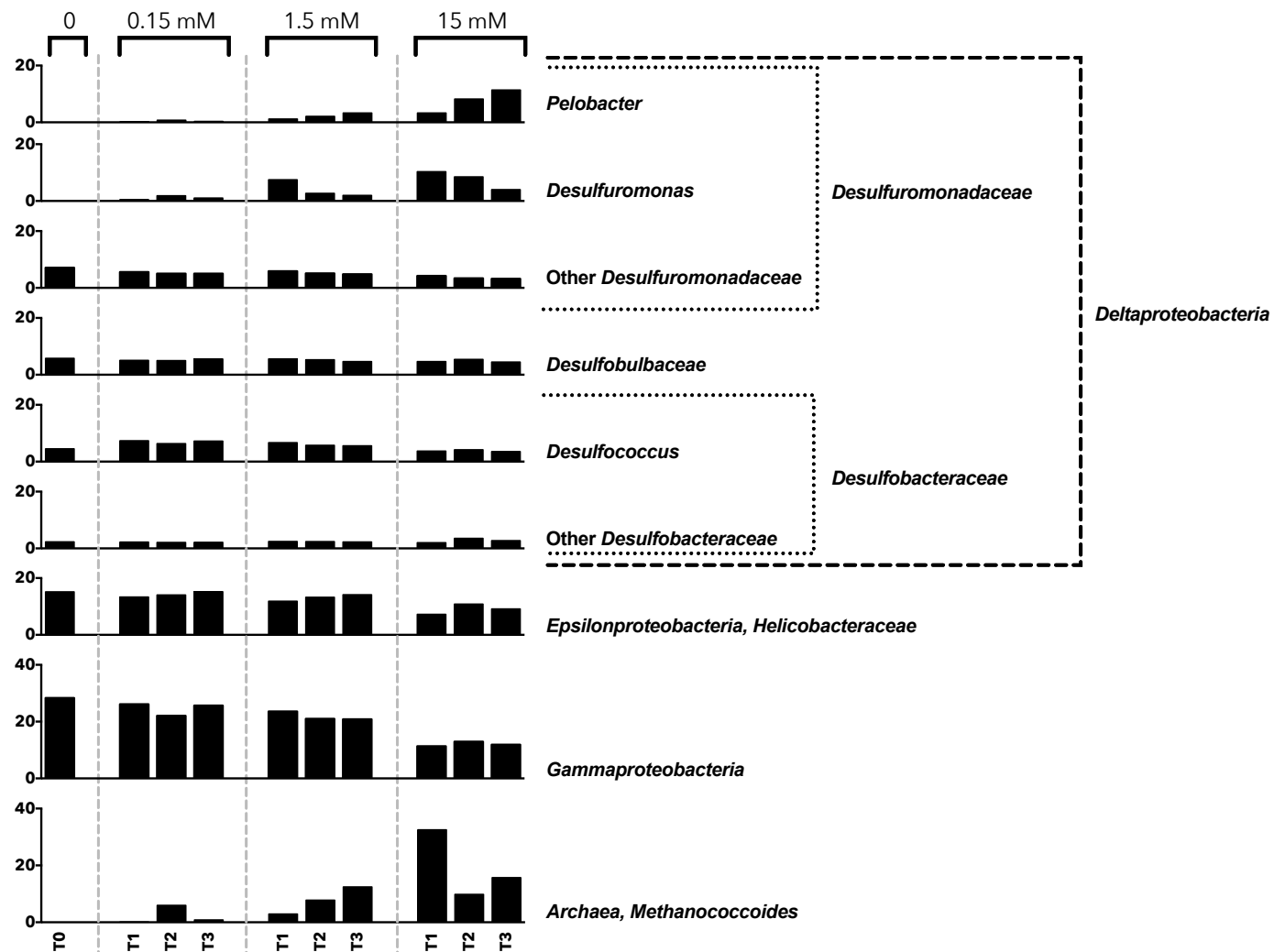

Supplement: Supplementary file 6 — Supplementary Figure [file 41396_2018_269_MOESM6_ESM.pdf]
